# Polypharmacy and potentially inappropriate prescribing of benzodiazepines in older nursing home residents

**DOI:** 10.1080/07853890.2024.2357232

**Published:** 2024-06-04

**Authors:** Ingrid Kummer, Jindra Reissigová, Anna Lukačišinová, Maja Ortner Hadžiabdić, Matej Stuhec, Rosa Liperoti, Harriet Finne-Soveri, Graziano Onder, Hein van Hout, Daniela Fialová

**Affiliations:** a Department of Social and Clinical Pharmacy, Faculty of Pharmacy in Hradec Králové, Charles University, Hradec Králové, The Czech Republic; b Department of Statistical Modelling, Institute of Computer Science of the Czech Academy of Sciences, Prague, The Czech Republic; c Center for Applied Pharmacy, Faculty of Pharmacy and Biochemistry, University of Zagreb, Zagreb, Croatia; d Department of Pharmacology, Faculty of Medicine Maribor, University of Maribor, Maribor, Slovenia; e Department of Clinical Pharmacy, Ormoz Psychiatric Hospital, Ormoz, Slovenia; f Fondazione Policlinico Universitario A. Gemelli IRCCS, Università Cattolica del Sacro Cuore, Rome, Italy; g National Institute for Health and Welfare, Helsinki, Finland; h Departments of General Practice and Medicine for Older People, Amsterdam University Medical Center, location Vrije Universiteit, Amsterdam Public Health Research Institute, Amsterdam, The Netherlands; i Department of Geriatrics and Gerontology, 1st Faculty of Medicine, Charles University, Prague, The Czech Republic

**Keywords:** Nursing home residents, polypharmacy/hyperpolypharmacy, psychiatric polypharmacy/hyperpolypharmacy, inappropriate benzodiazepine prescribing, geriatric deprescribing

## Abstract

**Introduction:**

Previous research has raised concerns about high prevalence of drug-related problems, polypharmacy and inappropriate benzodiazepine prescribing in nursing homes (NHs) and confirmed lack of studies from Central and South-Eastern Europe. The aim of our study was to determine the prevalence and characteristics of polypharmacy, hyperpolypharmacy and inappropriate benzodiazepine prescribing in NH residents in Croatia.

**Methods:**

Data from 226 older NH residents from five Croatian NHs were collected using the InterRAI Long-Term Care Facilities assessment form. The prevalence and determinants of polypharmacy/hyperpolypharmacy and patterns of inappropriate benzodiazepine prescribing were documented.

**Results:**

The prevalence of polypharmacy (49.6%) and hyperpolypharmacy (25.7%) among NH residents was high. In our study, 72.1% of NH residents were prescribed at least one psychotropic agent, 36.7% used 2–3 psychotropics and 6.6% used 4+ psychotropics. Among benzodiazepine users (55.8%), 28% of residents were prescribed benzodiazepines in higher than recommended geriatric doses, 75% used them for the long term and 48% were prescribed concomitant interacting medications. The odds of being prescribed polypharmacy/hyperpolypharmacy were significantly higher for older patients with polymorbidity (6+ disorders, proportional odds ratio (POR) = 19.8), type II diabetes (POR = 5.2), ischemic heart disease (POR = 4.6), higher frailty (Clinical Frailty Scale (CFS ≥5); POR = 4.3) and gastrointestinal problems (POR = 4.8).

**Conclusions:**

Our research underscores the persistent challenge of inappropriate medication use and drug-related harms among older NH residents, despite existing evidence and professional campaigns. Effective regulatory and policy interventions, including the implementation of geriatrician and clinical pharmacy services, are essential to address this critical issue and ensure optimal medication management for vulnerable NH populations.

## Introduction

The phenomena of polypharmacy, hyperpolypharmacy and the prescribing of potentially inappropriate medications (PIMs) are recognized as significant issues in geriatric prescribing, particularly in nursing homes (NHs) [1,2]. Nursing home residents are generally frail, have high levels of multimorbidity, use multiple medicines, have significant changes in pharmacokinetics and pharmacodynamics of used medications and as such are at high risk of prescribing cascades, polypharmacy, use of PIMs, adverse drug events and medication-related harms [3–6].

Polypharmacy, hyperpolypharmacy and prescribing of PIMs are all well documented in the NH setting. Previous research has reported that between 50 and 81% of older NH residents experience polypharmacy [4,5,7–10], up to 32% of residents experience hyperpolypharmacy and around 85% of residents have been prescribed one or more PIMs [11,12]. The most commonly prescribed PIMs in the NH setting are psychotropic medicines accounting for up to 73% of PIMs prescribed [13]. Psychotropic medications are commonly used by NH residents with studies estimating their prevalence up to 50% in total, for antidepressants by up to 75% and for benzodiazepines in some studies even up to 98.6% [14]. In addition to being one of the most often used medications among NH residents, a large proportion of benzodiazepines are considered to be unnecessarily or inappropriately prescribed in the NH setting. Previous studies reported that almost half of all benzodiazepines prescribed in NHs are considered inappropriate and new guidelines more strictly recommend not to exceed limits for geriatric indications, dosing, length of drug therapy and advice to carefully check all various drug interactions [9,15–17].

PIMs, polypharmacy and hyperpolypharmacy have all been shown to increase the risk of drug-related harm in the NH setting. A systematic literature review of 26 reviews across 230 studies found that polypharmacy increases the risk of impaired physical and cognitive functioning and self-performance, frailty and sarcopenia, poorer quality of life (QoL) and hospitalization and mortality [6]. Furthermore, polypharmacy has been found to have considerable ethical and economic negative impact [7]. Like polypharmacy and hyperpolypharmacy, use of PIMs and in particular, inappropriate prescribing of benzodiazepines, have been associated with increased mortality and morbidity in older age [18].

Like much of the world the Croatian population is aging, in 2019, 21% of the population was aged 65 years and older and this is expected to increase to 33% by 2070 [19]. With this aging population comes an increased need for high quality NH care and increased responsibility to ensure that preventable harms, such as those resulting from polypharmacy or the use of PIMs, are avoided. Currently in Croatia, NHs use a model of care based on NH-based nursing staff and visiting general practitioners. Despite research showing the value of medication review in improving the quality of medicine use in the NH setting [20], Croatian NH residents do not have access to onsite geriatrician or clinical pharmacy services [21,22].

While the prevalence of polypharmacy, hyperpolypharmacy and PIMs has been well researched in Western Europe and Northern America, much less is known about the extent of these problems in Central and South-Eastern Europe, and particularly in the NH setting. A study of 200 NH residents in South West Slovenia found that benzodiazepines were the most commonly prescribed PIMs, representing 50% of all PIMs prescribed to study participants [11]. In this study, it was noted that key problems associated with the use of benzodiazepines by NH residents were related to the use for inappropriate indications and extended durations [11]. It was also reported that inappropriate benzodiazepine use could be reduced by clinical pharmacist review and intervention [11]. A previous study on 73 Croatian NH residents found that almost three quarters of residents were prescribed polypharmacy and that on average researchers found 4.3 drug-related problems identified per residents [23], indicating considerable need for research to understand and improve medication prescribing and use practices in Croatia. However, this study was relatively small and with 11,000 NH residents currently residing in Croatia [21,22], deeper understanding of prescribing practice and appropriateness of medicine use is needed.

Avoiding geriatric polypharmacy, hyperpolypharmacy and PIM use is a real challenge, requiring detailed geriatric pharmacotherapy and pharmacological knowledge, advanced clinical skills in geriatric medication reviews, and individualized approaches to optimize combined drug regimens. Up to date, there has been limited research exploring the extent of polypharmacy, hyperpolypharmacy and prescribing of PIMs in NH population in Croatia, as well as in other Central and South-Eastern European countries. Understanding the extent of polypharmacy, hyperpolypharmacy and PIMs use in Croatian NHs and the factors contributing to them is the first step in determining strategies that might help in reducing drug-related harm in this vulnerable older population. Therefore, the aim of our research was to determine the prevalence and characteristics of polypharmacy, hyperpolypharmacy and potentially inappropriate benzodiazepine use among NH residents in Croatia.

## Materials and methods

### Study design and sampling

This multicentre cross-sectional study was conducted between 2 August 2022 and 12 December 2022, in 226 NH residents aged 65 years and older in five NHs in Croatia. Nursing homes included in the study were selected to be from different geographical regions (City of Zagreb, Slavonia and Dalmatia).

The data collection and preliminary analyses were conducted within the START project, supported by the European Structural and Investment Funds – Operational program ‘Research, Development and Education’ at the Charles University, Czech Republic. The START project aimed to describe the current situation and inappropriate prescribing patterns in NHs in four European countries (Czech Republic, Croatia, Bulgaria and Slovakia). The final analyses and publication of results were also supported by the project NETPHARM/New Technologies for Translational Research in Pharmaceutical Sciences (project ID CZ.02.01.01/00/22_008/0004607), co-funded by the European Union.

### Recruitment

A convenience sample of NH residents 65+ from five NHs participated in the study. The study sample was not randomly selected and was not intended to be representative of all NH residents in Croatia.

Residents from participating NHs who met the inclusion criteria and gave their informed consent were included in the study. The inclusion criteria were: age ≥65 years, duration of NH residence ≥3 months and no intensive care. Exclusion criteria were severe dementia defined as a Mini-Mental State Examination (MMSE) score <10, terminal illness or palliative care (life expectancy <12 months), serious communication disorders (e.g. serious problems with hearing or speaking with no corrective device) or refusal to consent to participation in the study. Patients acutely hospitalized or visiting emergency departments at the time of researchers’ visit of a NH could not be assessed but were interviewed later than 1 week after return to the NH (after their health status was stabilized) if they signed the informed consent to participate in the study.

### Data collection tools and processes

Study data were obtained via interviews with NH residents, and information was clarified from medical charts and/or by interview with physicians and/or caring nurses.

Data were collected using the *InterRAI Long Term Care Assessment Form (InterRAI LTCF)* [24]. The InterRAI LTCF is a standardized and validated comprehensive geriatric assessment tool. This instrument was translated from English by two independent translators into the Croatian language and then back-translated into English to ensure translation consistency. The translated version was then piloted on 10 NH residents.

Within the InterRAI LTCF characteristics, a functional status of geratric patients is assessed using standardized and validated scales embedded within the InterRAI LTCF tool [25]. In our study were used the Depression Rating Scale (DRS) [26]; Cognitive Performance Scale (CPS) [27], and the Pain Scale [28]. The assessment by Clinical Frailty Scale (CFS) was added [29] Diagnoses were coded as current diagnoses (receiving active treatment or monitored) using diagnostic categories specifically stated in the InterRAI LTCF form. While major diagnoses were predefined in the InterRAI LTCF tool, other specific diagnoses were added using the International Classification of Disorders coding (ICD-11 Version: 2020) (see Supplementary material) [30].

Data collected for each medication included: trade name, active ingredient, the Anatomical-Therapeutic-Chemical (ATC) classification code [31], pharmaceutical form, the strength of one dosage unit (e.g. one tablet), dosing regimen and duration of treatment. Data for all medications used in the past seven days prior to the date of data collection were captured. Data were also captured on medications used in other than daily regimens, e.g. once or twice a month or every six months (e.g. bisphosphonates, vitamin D, methotrexate, etc.). Vitamins, dietary supplements and herbal products were recorded as well, but did not have ATC codes, so were analysed as one product (each). Information on regular use or the use of drugs as-needed (PRN (*pro re nata*)) was also recorded. The last medication-related characteristic captured was the length of drug therapy for each medication used (obtained from the healthcare records or estimated by the NH resident).

Research staff were trained in the use of the InterRAI LTCF tool, including interpretation, scoring and recording of the items.

### Main outcome measures and definitions

The objectives of our study were to determine current prevalence of polypharmacy/psychotropic polypharmacy, hyperpolypharmacy/psychotropic hyperpolypharmacy and benzodiazepine prescribing and to explore characteristics of each of these.

Polypharmacy was defined as the concurrent use of 5–9 medications, and hyperpolypharmacy as the use of ≥10 medications concurrently [4,7–9]. We analysed these prevalence by calculating drug products, but also by calculating active substances (vitamins, dietary supplements and herbal products were always calculated as one drug product even if they included multiple active substances).

Psychotropic polypharmacy was defined as the use of 2–3 psychotropics (including benzodiazepines) and psychotropic hyperpolypharmacy as concurrent use of four or more psychotropics (including benzodiazepines) [32]. Psychotropic medicines were all medicines included in ATC classes N02A, N04, N05A, N05B, N05C, N06A, N06BA, N06BC, N06C and N06D (see also Supplementary material).

Benzodiazepines were defined as all medications having ATC codes N05BA or N05CD and both regular and PRN use were captured.

PIMs were defined as all medicines listed in the Beers criteria ver. 2019 [33], STOPP/START ver. 2 criteria [34] and EU(7)-PIM list [35] as well as medicines listed in other geriatric guidelines and studies (see also legend of the Supplementary Table 1).

Potentially inappropriate benzodiazepine use was considered: (1) the use of benzodiazepines in a single dose higher than the recommended geriatric single dose (at any time during the day); (2) the use of a higher daily dose of benzodiazepines than recommended for geriatric patients, (3) use of benzodiazepines for >4 weeks (according to current geriatric guidelines this threshold should not be exceeded in any diagnosis (except drug dependence that cannot be clarified from our data)), (4) the concurrent use of other drugs in the drug regimen that may cause a drug interaction of a moderate to severe clinical significance with benzodiazepines (Medscape [36] and DrugBank Online databases [37] were used for the drug interaction search, most often used sources in Croatia) and all interactions labelled moderate to severe by significance were recorded (see also Supplementary Table 3), (5) concurrent use of benzodiazepines with other sedative medications, (6) use of psychotropic polypharmacy and (7) use of benzodiazepines only for the diagnosis of insomnia (instead of more appropriate Z-drugs or alternative safer drug treatments, such as trazodone and mirtazapine). Since the intended indication was not clarified in the residents’ medication records, we assumed that if a resident had only a diagnosis of insomnia and used benzodiazepine once daily in the evening or at bedtime, the main indication was most probably the treatment of insomnia.

### Sample size

A sample of 226 NH residents was determined to be sufficient to estimate the prevalence of inappropriate prescribing to obtain a 95% confidence interval (CI) with a margin of error of 6.5% (i.e. with accuracy ±6.5%) when 50% prevalence of polypharmacy was conservatively assumed.

### Statistical analyses

Numeric variables were presented as mean ± standard deviation (SD) or median with interquartile range (IQR, lower–upper quartile). Categorical variables were described by the absolute frequencies and percentages. The prevalence of benzodiazepines was expressed as a percentage and the 95% CI was calculated using the Clopper–Pearson method. Numeric variables among the groups (no polypharmacy, polypharmacy, hyperpolypharmacy) were compared using analysis of variance (ANOVA) or Kruskal–Wallis test if assumptions of ANOVA (normality, homoscedasticity) were not met. Spearman’s correlation (*r*s) was performed to quantify the relationship between two numeric variables. The chi-square or Fisher’s test (if not all expected counts were at least 5) was used to explore whether the distribution of categorical variables is homogeneous across the groups.

**Figure 1.**
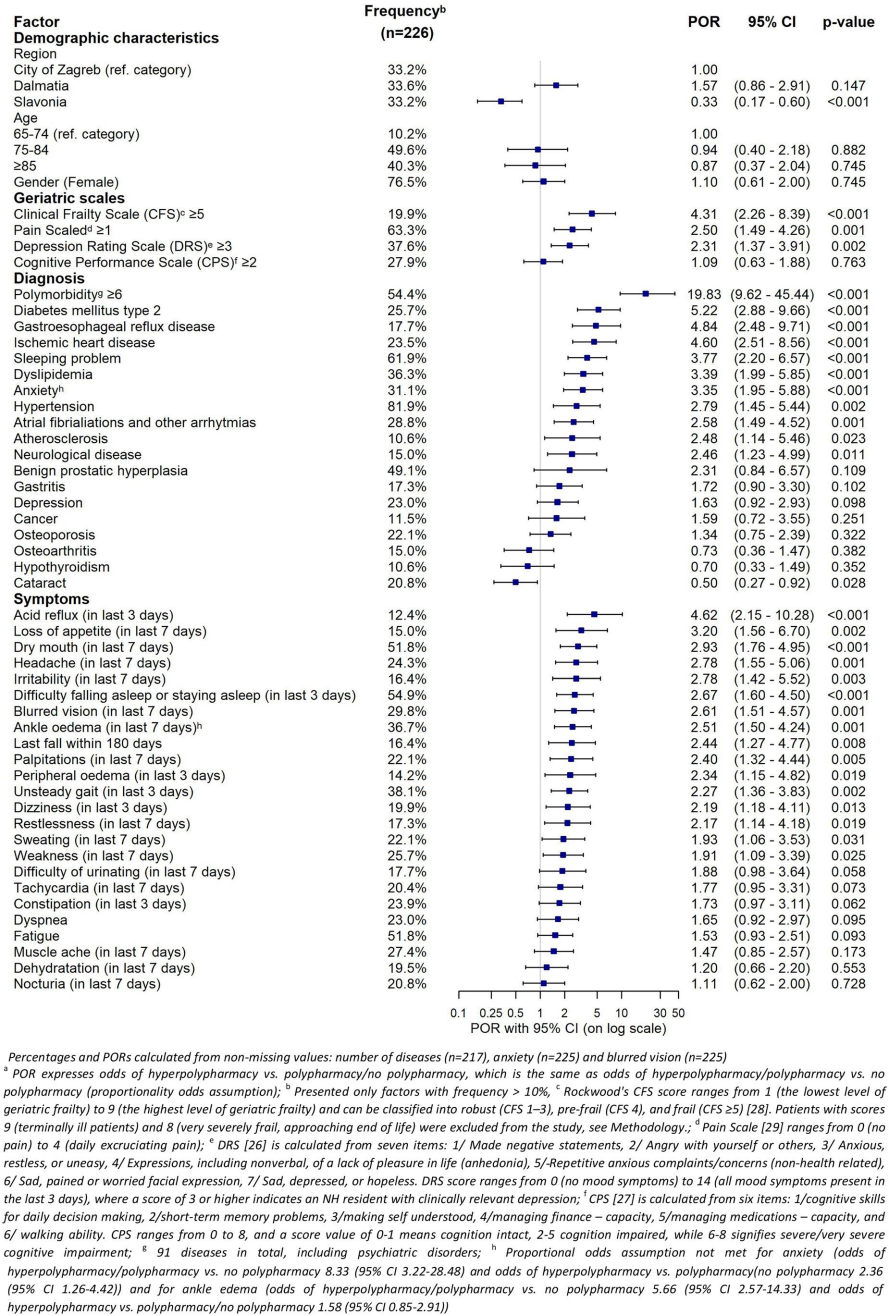
The strength of association between a higher number of drugs and potentially predictive factors (POR, proportional odds ratio^a^).

Ordinal (univariable) logistic regression was used to estimate the strength of the association between a higher number of drugs and potential predictive factors (Figure 1). The strength of the association was reported using the proportional odds ratio (POR) with 95% CIs (profile likelihood CI). Proportional OR means that the odds ratios (ORs) are the same at each cumulative split of the ordinal dependent variable (no polypharmacy, polypharmacy, hyperpolypharmacy). In case of our study, it means that the odds of hyperpolypharmacy versus polypharmacy or no polypharmacy were the same as the odds of hyperpolypharmacy or polypharmacy versus no polypharmacy. The proportional odds assumption was tested using Brant’s test and graphically examined. If the assumption of proportional odds was not met, separate binary logistic regressions were applied to estimate the (non-proportional) ORs. The alpha level was set at .05 for statistical significance. Data were analysed using the R software version 4.2.2 (R Foundation for Statistical Computing, Vienna, Austria) [38].

### Ethical approval

The study was approved by the Ethics Committee of the Faculty of Pharmacy in Hradec Králové, Charles University, Hradec Králové, Czech Republic (number UKFaF/297850/2022). Participants were informed about the intention of the study, objectives and outcomes prior to recruitment and all participants received written participant information sheets. Data were collected and stored anonymously using specific study codes and the study followed the European Union General Data Protection Regulation (EU GDPR) anonymity and confidentiality rules.

## Results

Two hundred and twenty-six residents from five NHs were included in this study, with the mean age of 82.5 ± 6.2 years, 89.8% were in the age group 75+ and the majority were women (76.5%). A total of 63.3% of seniors reported pain (Pain Scale ≥1), 27.9% were cognitively impaired (CPS ≥2) and 37.6% suffered from clinically significant depression (DRS ≥3). Nearly, 20% of seniors were in higher stages of geriatric frailty (CFS ≥5).

### Polypharmacy and hyperpolypharmacy

Of the 226 NH residents assessed in this study, 49.6% had polypharmacy and 25.7% hyperpolypharmacy (using calculations based on number of drug products). According to the number of active substances (where combination vitamins, herbal products and dietary supplements not having ATC code were considered as a single active ingredient), polypharmacy was found to be 46.9% and hyperpolypharmacy 33.6%.

There was a strong association between the number of drugs used and the number of disorders diagnosed (*r*s = 0.74, *p* < .001). Residents with polymorbidity (≥6 disorders) had 19.8 times higher odds of receiving polypharmacy or hyperpolypharmacy. Type 2 diabetes mellitus, gastroesophageal reflux and ischemic heart disease were disorders most significantly associated with a higher number of medications used (POR >4.5) (Figure 1). Residents prescribed polypharmacy or hyperpolypharmacy were more likely to have anxiety, sleeping problems and neurological disorders (see more results in Table 1 and Figure 1).

**Table 1.** Main characteristics of nursing home residents.

Characteristic	Number of medications								
	Total (*n* = 226)		No polypharmacy 1–4 (*n* = 56)		Polypharmacy 5–9 (*n* = 112)		Hyperpolypharmacy ≥10 (*n* = 58)		*p*-value
Region									
City of Zagreb	33.2%	(75)	19.6%	(11)	41.1%	(46)	31.0%	(18)	<.001
Dalmatia	33.6%	(76)	23.2%	(13)	28.6%	(32)	53.4%	(31)	
Slavonia	33.2%	(75)	57.1%	(32)	30.4%	(34)	15.5%	(9)	
Age, years mean ± SD	82.5 ± 6.2		82.1 ± 6.0		82.8 ± 6.5		82.2 ± 5.8		.732
Age categories									
65–74	10.2%	(23)	8.9%	(5)	10.7%	(12)	10.3%	(6)	.367
75–84	49.6%	(112)	55.4%	(31)	42.9%	(48)	56.9%	(33)	
≥85	40.3%	(91)	35.7%	(20)	46.4%	(52)	32.8%	(19)	
Gender (female)	76.5%	(173)	71.4%	(40)	80.4%	(90)	74.1%	(43)	.385
Number of diseases, median (IQR)	6 (4–8)		3 (2–5)		6 (5–8)		9 (7–13)		<.001
Number of diseases^a^									
0–3	18.4%	(40)	58.5%	(31)	8.2%	(9)	0.0%	(0)	<.001
4–5	27.2%	(59)	35.8%	(19)	30.9%	(34)	11.1%	(6)	
≥6	54.4%	(118)	5.7%	(3)	60.9%	(67)	88.9%	(48)	
Psychiatric disorders									
Sleeping problem	61.9%	(140)	33.9%	(19)	67.9%	(76)	77.6%	(45)	<.001
Anxiety	31.1%	(70)	7.1%	(4)	35.7%	(40)	45.6%	(26)	<.001
Depression	23.0%	(52)	14.3%	(8)	25.0%	(28)	27.6%	(16)	.188
Neurological diseases^b^	15.0%	(34)	7.1%	(4)	14.3%	(16)	24.1%	(14)	.038
Dementia	4.0%	(9)	1.8%	(1)	4.5%	(5)	5.2%	(3)	.745
Panic disorders	1.8%	(4)	0.0%	(0)	1.8%	(2)	3.4%	(2)	.466
Geriatric scales									
Depression Rating Scale (DRS)^c^ ≥3	37.6%	(85)	25.0%	(14)	35.7%	(40)	53.4%	(31)	.006
Cognitive Performance Scale (CPS)^d^ ≥2	27.9%	(63)	26.8%	(15)	27.7%	(31)	29.3%	(17)	.954
Pain Scale^e^ ≥1	63.3%	(143)	48.2%	(27)	62.5%	(70)	79.3%	(46)	.003
Clinical Frailty Scale (CFS)^f^ ≥5	19.9%	(45)	7.1%	(4)	16.1%	(18)	39.7%	(23)	<.001
Symptoms									
Chronic pain	54.9%	(117)	41.5%	(22)	56.2%	(59)	65.5%	(36)	.041
Fatigue	51.8%	(117)	44.6%	(25)	50.9%	(57)	60.3%	(35)	.237
Unsteady gait (in last 3 days)	38.1%	(86)	21.4%	(12)	40.2%	(45)	50.0%	(29)	.006
Headache (in last 7 days)	24.3%	(55)	12.5%	(7)	22.3%	(25)	39.7%	(23)	.003
Dyspnoea	23.0%	(52)	16.1%	(9)	23.2%	(26)	29.3%	(17)	.244
Dizziness (in last 3 days)	19.9%	(45)	10.7%	(6)	19.6%	(22)	29.3%	(17)	.045
Loss of appetite (in last 7 days)	15.0%	(34)	8.9%	(5)	10.7%	(12)	29.3%	(17)	.002
Hypotension (in last 7 days)	11.1%	(25)	10.7%	(6)	10.7%	(12)	12.1%	(7)	.961
Abnormal thought process (in last 3 days)	5.8%	(13)	3.6%	(2)	7.1%	(8)	5.2%	(3)	.758
Bradycardia (in last 7 days)	3.5%	(8)	0.0%	(0)	3.6%	(4)	6.9%	(4)	.134
Syncope (in last 7 days)	0.4%	(1)	0.0%	(0)	0.0%	(0)	1.7%	(1)	.504
Prevalence of drugs									
Psychotropic meds 1+ (incl. BZD)	72.1%	(163)	37.5%	(21)	77.7%	(87)	94.8%	(55)	<.001
BZDs	55.8%	(126)	23.2%	(13)	60.7%	(68)	77.6%	(45)	<.001
Opioids	31.0%	(70)	7.1%	(4)	31.2%	(35)	53.4%	(31)	<.001
Hypnotics (excl. BZD)	17.7%	(40)	8.9%	(5)	12.5%	(14)	36.2%	(21)	<.001
Antidepressants	13.7%	(31)	5.4%	(3)	15.2%	(17)	19.0%	(11)	.088
Antipsychotics	10.6%	(24)	0.0%	(0)	9.8%	(11)	22.4%	(13)	<.001
Antiparkinsonics	1.8%	(4)	0.0%	(0)	2.7%	(3)	1.7%	(1)	.809
Antidementia medications	0.9%	(2)	0.0%	(0)	0.0%	(0)	3.4%	(2)	.126
Anticholinergic medications	80.1%	(181)	44.6%	(25)	87.5%	(98)	100.0%	(58)	<.001
Medications with sedative properties	32.7%	(74)	12.5%	(7)	28.6%	(32)	60.3%	(35)	<.001

Percentages calculated from non-missing values: missing values were found in number of diseases (*n* = 217), particularly for anxiety (*n* = 225) and chronic pain (*n* = 213).

^a^ Ninety-one diseases analysed.

^b^ Neurological diseases include Alzheimer’s disease, vascular dementia, Lewy body dementia, other dementia, multiple sclerosis, traumatic brain injury (TBI), Parkinson’s disease, stroke (CVA), quadriplegia, paraplegia and hemiplegia.

^c^ DRS [26] is calculated from seven items: (1) made negative statements, (2) angry with yourself or others, (3) anxious, restless or uneasy, (4) expressions, including nonverbal, of a lack of pleasure in life (anhedonia), (5) repetitive anxious complaints/concerns (non-health related), (6) sad, pained or worried facial expression and (7) sad, depressed or hopeless. DRS score ranges from 0 (no mood symptoms) to 14 (all mood symptoms present in the last three days), where a score of 3 or higher indicates a NH resident with clinically relevant depression.

^d^ CPS [27] is calculated from six items: (1) cognitive skills for daily decision making, (2) short-term memory problems, (3) making self-understood, (4) managing finance – capacity, (5) managing medications – capacity and (6) walking ability. CPS ranges from 0 to 8, and a score value of 0–1 means cognition intact, 2–5 cognition impaired, while 6–8 signifies severe/very severe cognitive impairment.

^e^ Pain Scale [29] ranges from 0 (no pain) to 4 (daily excruciating pain).

^f^ Rockwood’s CFS score ranges from 1 (the lowest level of geriatric frailty) to 9 (the highest level of geriatric frailty) and can be classified into robust (CFS 1–3), pre-frail (CFS 4) and frail (CFS ≥5) [28]. Patients with scores 9 (terminally ill patients) and 8 (very severely frail, approaching end of life) were excluded from the study, see ‘Materials and methods’ section.

### Psychotropic medication use

Of all residents, 72.1% in this sample used one or more psychotropic drug. The prevalence of use of the different drug groups was: benzodiazepines (55.8%), opioids (31.0%), hypno-sedatives (excluding benzodiazepines (17.7%)), antidepressants (13.7%) and antipsychotics (10.6%).

Residents with polypharmacy or hyperpolypharmacy were more likely to use psychotropic medications, medication with anticholinergic properties, and sedative medications (*p* < .001 for all cases), but compared groups did not differ significantly in age or sex (see Table 1).

### Potentially inappropriate use of benzodiazepines

**Figure 2.**
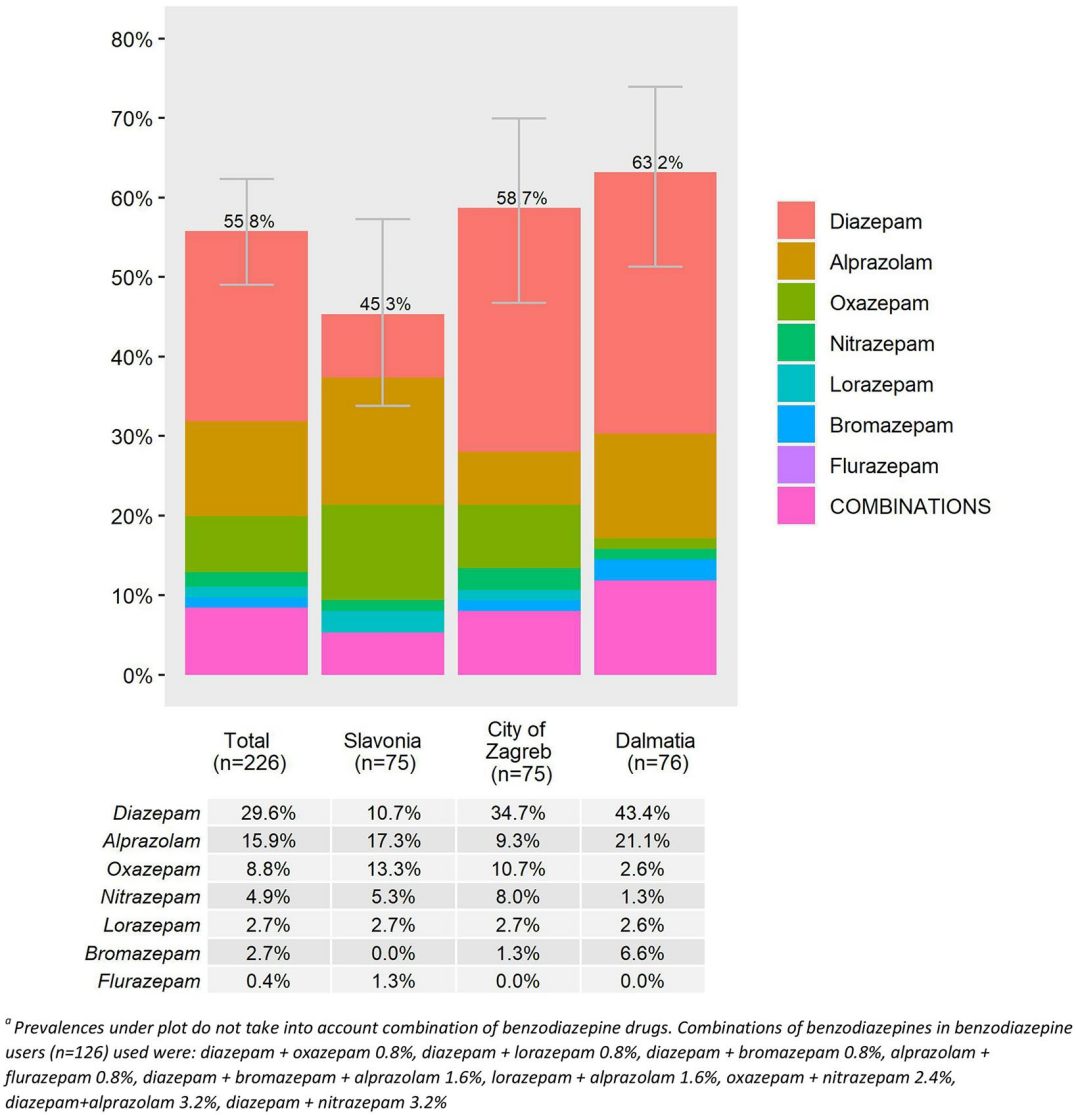
Prevalence of benzodiazepine use in nursing home residents^a^.

In this sample, 55.8% (*n* = 126) of residents were prescribed one or more benzodiazepines, and 8.4% used two or more benzodiazepines simultaneously (Figure 2). We observed regional differences (*p* = .073) in the prevalence of benzodiazepine use with the highest prevalence in Dalmatia (63.2%), and the lowest in Slavonia (45.3%) (Figure 2). Diazepam (29.6%), alprazolam (15.9%) and oxazepam (8.8%) were the most frequently prescribed benzodiazepines and 63.5% of all benzodiazepines were used regularly rather than PRN.

**Figure 3.**
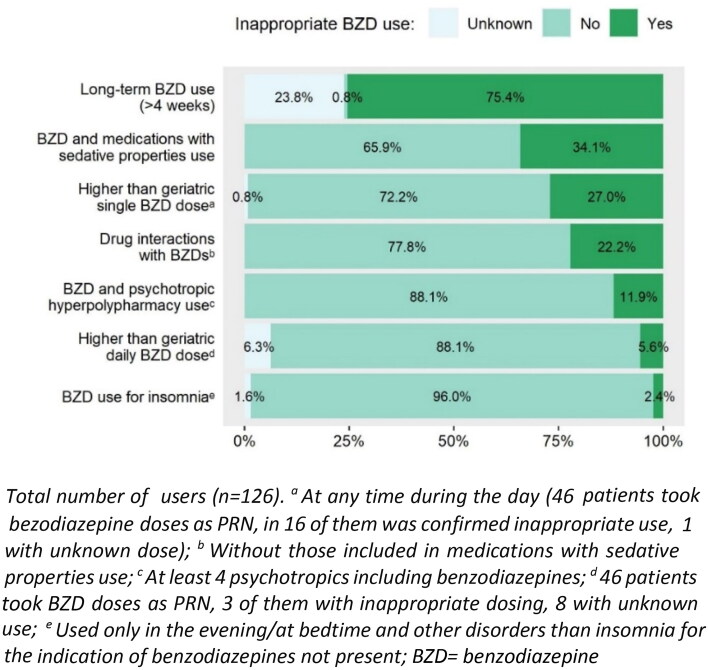
Potentially inappropriate benzodiazepine prescribing in nursing home residents.

When we analysed different patterns of potentially inappropriate use of benzodiazepines (see Figure 3), the use of a single dose higher than the recommended geriatric dose (1) was identified in 27.0% of benzodiazepine users and use of a higher than recommended daily geriatric dose in 5.6% of users; (2) length of the treatment for more than 4 weeks in 75.4% of users; (3) and combinations of benzodiazepines with other sedative medications (only 1 sedative drug) used 27% of NH residents (see Figure 3) and 7.1% of residents were reusing a benzodiazepine with two or more other sedatives, most commonly with zolpidem (in 15.9% of cases), followed by sedative antihistamines for systemic use (see also Supplementary Table 2). Prescribing of drugs most probably significantly interacting with benzodiazepines (4) was determined in 14.3% of benzodiazepine users (only drug interaction with a moderate to severe clinical significance were analysed) and 7.9% of users were prescribed two or more such interactions. The most prevalent drug interaction (present in 10.3% of benzodiazepine users) was the combination of alprazolam/diazepam/lorazepam/oxazepam and moxonidine, the combination of diazepam/nitrazepam and paracetamol (in 2.4% of benzodiazepine users) and the interaction of alprazolam and metildigoxin (also in 2.4% of users). The use of benzodiazepines with psychotropic polypharmacy/hyperpolypharmacy was observed in 53.2% and 11.9% of users of these medications, respectively. Looking in detail at psychotropic polypharmacy, the combination of benzodiazepine with tramadol/paracetamol was as the most often prescribed combination (in 50.6% of users).

Overall, 54.0% of benzodiazepines users met at least one criterion of potentially inappropriate benzodiazepines prescribing (excluding missing data and long-term use that could not be clarified in many subjects).

## Discussion

In this study, we found that polypharmacy, hyperpolypharmacy and potentially inappropriate use of benzodiazepines were common among Croatian NH residents. Potentially inappropriate geriatric prescribing is highly interconnected in various clinical situations with undesirable outcomes [5], which is highlighting the need for strategies to reduce PIMs, polypharmacy and increase safer medication prescribing practices. We focused on describing of the current situation regarding polypharmacy and benzodiazepine prescribing in complex older residents in NHs in Croatia, a country in South-East Europe with a high prevalence of benzodiazepine utilization according to previous studies [39]. In addition, we presented a theoretical construct for identifying specific patterns of potentially inappropriate benzodiazepine prescribing.

The prevalence of polypharmacy and hyperpolypharmacy in our sample was high with about half of the residents in our sample prescribed 5–9 medicines concurrently and one quarter prescribed 10 or more medicines. Our findings are similar to that noted in other studies indicating that polypharmacy and hyperpolypharmacy among NH residents truly is an international problem and that innovative strategies are needed to reduce the burden of polypharmacy in our older NH populations [4,7,9,10]. Our sample was similar to those reported in NHs internationally. Almost 40% of the residents in our sample were diagnosed with minor to major depressive problems, 30% suffered from cognitive impairment and 20% were assessed as residents in higher stages of geriatric frailty. All these characteristics correspond with findings in other published studies, for example, in Portugal, the Czech Republic, England, Finland, France, Germany, Italy, the Netherlands and Israel [5,8,9]. We identified a wide range of characteristics associated with polypharmacy and hyperpolypharmacy. Particularly frailty, higher pain scores and higher levels of depression were all associated with polypharmacy and hyperpolypharmacy in our research which is similar to findings reported in the SHELTER study conducted in 57 European NHs [9].

Psychotropic polypharmacy and psychotropic hyperpolypharmacy also occurred frequently in our study population with over one third of residents using two to three psychotropic agents concurrently. Psychotropic polypharmacy in our sample was considerably higher than reported previously. A study of 18,853 residents across 90 NHs in Switzerland found that psychotropic polypharmacy was experienced by approximately 10% of residents [40] while a study of 4739 residents from 129 Norwegian homes found that approximately one quarter (23.4%) of residents had psychotropic polypharmacy [41]. Our findings indicate a high need for strategies to optimize psychotropic use in Croatian NHs with optimal psychotropic prescribing that require prescribing for appropriate indications with individualization dosing to meet each individual’s needs [42].

The most prescribed psychotropic drugs in our population were benzodiazepines which were used by more than half of all residents included in our study. Similar findings have been reported in other NH studies internationally with estimates ranging from 30.5% to 54.5% [9,15–17]. We observed regional variation in the use of benzodiazepines across the three geographical regions. Similar regional variation has been reported in Norway and it is estimated that interregional social and behavioural factors could contribute to these differences [14]. Another contributing factor that could explain the high prevalence of potentially inappropriate benzodiazepine prescribing and regional variations is the absence of national guidelines on rational benzodiazepine prescribing in geriatric patients in Croatia [39]. In the EuroAgeism H2020 project (2017–2022) conducted in older patients visiting pharmacies in eight European countries, the exposure to benzodiazepines in Croatia was the highest (35.5%) across all included countries (with the lowest prevalence in Turkey, 0.7%) [43]. The EuroAgeism project [44] aimed to identify the most common prescribing problems in older adults in Central and South-Eastern Europe (in the Czech Republic, Estonia, Serbia, Croatia, Bulgaria, Turkey and Spain) to support the need for availability of clinical pharmacy services. Another ongoing European project, the I-CARE4OLD H2020 project (2021–2025) aims to prepare computerized algorithms (by using artificial intelligence methods) to better estimate the appropriate indications of various pharmacological and non-pharmacological interventions in older adults with complex comorbid conditions in NHs and home care [45]. The classification of inappropriate benzodiazepine use proposed in this research provides one such construct that could be used with digital or artificial intelligence methods for identifying residents in need of benzodiazepine deprescribing services in the future.

In our study, the three most frequently prescribed benzodiazepines were diazepam, alprazolam and oxazepam. According to the Annual Report on Drug Use in Croatia in 2022, diazepam and alprazolam were among the top 10 medications most often used in this country [46]. The EuroAgeism project reported similar patterns to those that we observed among NH older persons, with diazepam being the most prescribed benzodiazepine in Croatia also among community-dwelling older adults [43]. A national study using claims data confirmed that benzodiazepine prescriptions increased by 4.1% in Croatia between 2015 and 2016 [39]; therefore, strategies to improve the appropriateness of benzodiazepine prescribing in Croatia appear to be needed.

In this study, we were able to characterize different elements comprising inappropriate benzodiazepine use. A maximum duration of 4 weeks, irrespective of the indication, is recommended for the safe use of these medications when treating older patients [47] yet in three quarter of the benzodiazepine users we identified the length of use >4 weeks. Similarly, lower doses are recommended for the use in older populations [48]; however, almost 30% of the users in our sample were taking a single dose higher than the recommended geriatric single doses. These findings highlight the need for implementing clinical pharmacy and geriatric services in Croatian NHs to improve the rational use of benzodiazepines.

This study emphasizes the need to reduce polypharmacy and inappropriate drug use in older NH residents in Croatia. Multifaceted approaches targeting different aspects of NH care and medication use should be considered. The Haute Autorité de Santé in France have released detailed guidelines on how to assist patients in discontinuing benzodiazepines [49] and there is a clear need for national Croatian guidelines for NHs and prescribers guidelines on appropriate prescribing and deprescribing of benzodiazepines. Implementation of other measures, many were already shown to be successful in improving older patients’ pharmacotherapy management in international settings, such as regulatory policy interventions, and higher availability of geriatric and clinical pharmacy services, should also be considered [50–52].

Like in many countries NHs in Croatia are often understaffed [21]. General practitioners usually visit NH residents once a week or less, and clinical pharmacists or consultant geriatricians are not regularly available onsite. Based not only on our experience from this conducted research, but also from daily clinical experience, common laboratory tests are not performed as often as needed, and medicine-related data is often out of date or incomplete. With ADEs contributing to poor physical and cognitive frailty, decline in QoL, and increased morbidity, mortality and healthcare costs among NH residents, reduction in drug-related harms and inappropriate medication prescribing is critical [53] and increased access to consulting geriatricians and clinical pharmacists a key element in reducing medication-related harm [54]. Research in France has demonstrated the cost-effectiveness of clinical pharmacy services in the NH setting finding a saving of over 200 euro per resident after implementing these services [50], providing a compelling case for the consideration of such services also in the Croatian context.

With the knowledge that multifaceted approaches are the most successful at changing prescribing behaviours and medication use [55], safer non-­pharmacological and evidence-based options can also help prescribers to reduce frequent benzodiazepine use [56]. Educational strategies and public health campaigns in isolation often failed to change prescribing behaviour in the longer term [57] and combined approaches including regulatory measures such as audit and feedback and policy measures should be considered, as well as restrictions on the maximum period of use for all benzodiazepines, and development of and access to benzodiazepine deprescribing services. Further research is needed to better understand the strong nonclinical factors that play a role in the appropriateness of benzodiazepine prescribing, e.g. habits, and social, cultural, economic and behavioural factors [58].

### Strengths and limitations

Like all research, this study had several strengths and limitations. In this study, we used the InterRAI LTCF assessment tool, a structured and validated international instrument for comprehensive geriatric assessments in NH residents, embedding various standardized geriatric scales and assessments with comprehensive information on medication use. Furthermore, this is one of the first studies to explore polypharmacy and inappropriate benzodiazepine use in Central and South-East European country which is a key strength of the paper. A limitation of the study is the use of a convenience sample for selecting the NHs to participate in this study and as a result we are unable to ensure that the study results fully represent the national situation. The smaller sample size did not allow us to apply multivariable regression to detect independent predictors of higher number of medications and therefore we generated univariable PORs (see Figure 1). As NHs are one of the most challenging settings of care for collecting research data and our study was conducted intentionally in different regions of Croatia, we had to overcome many research barriers often associated with research in the NH setting, but we were able to reach the planned study sample and collect data across three distinct geographical regions. Residents were highly willing to participate in our study and our low refusal rate (less than 5%) increases the information value of our results. A second limitation of this research was the use of a cross-sectional study design that did not enable to draw causal conclusions if some factors significantly associated with polypharmacy or hyperpolypharmacy in Croatian NHs are rather predictive factors or negative outcomes.

## Conclusions

Despite well respected evidence regarding the relationship between polypharmacy and inappropriate medication use and drug-related harms among older NH populations, our research shows that there is an ongoing need for strategies to reduce inappropriate prescribing in the NH setting, particularly in Central and South-East Europe. Even if international guidance is available on appropriate prescribing of benzodiazepines and long-term professional campaigns were ongoing to reduce unnecessary polypharmacy, hyperpolypharmacy and inappropriate benzodiazepine use in complex older adults, our findings show that polypharmacy and inappropriate benzodiazepine use continue to place a large proportion of our vulnerable older NH populations at an unacceptable risk of drug-related harms. Effective regulatory, policy and other multifaceted interventions are needed to ensure better medication management in the NH setting. In the Croatian context, the implementation of geriatric and clinical pharmacy services to support optimal medication management for all NH residents should be also considered.

## Supplementary Material

Supplemental Material

## Data Availability

The datasets used and/or analysed during the current study are available from the corresponding author as the chair of the research works in the START/MED/093 project called ‘Grant Schemes at Charles University’ (Reg. No. CZ.02.2.69/0.0/0.0/19_073/0016935) and the NETPHARM project/New Technologies for Translational Research in Pharmaceutical Sciences (project ID CZ.02.01.01/00/22_008/0004607).
