# Supplementary material for: Polypharmacy and potentially inappropriate prescribing of benzodiazepines in older nursing home residents
Source: Ann Med. 2024 Jun 4;56(1):2357232. doi: 10.1080/07853890.2024.2357232 (PMC11151799; doi:10.1080/07853890.2024.2357232)
Supplement: Supplemental Material [file IANN_A_2357232_SM6887.docx]

**Major diagnoses predefined in the interRAI LTCF tool**

*-by interRAI tool definition these are:*

*major conditions: conditions (common or otherwise) that influence the care required, the trajectory of care need, and life expectancy, conditions that require active treatment and that if not treated lead to major morbidity, suffering, or death, conditions that if not managed correctly may lead to harm to others (e.g., infectious diseases) and recent acute conditions that will require specific care in the subsequent 3 months*

*List of these predefined diagnoses in interRAI LTCF tool:*

- musculoskeletal diagnoses (hip fracture, other fracture during last 30 days); neurological – dementia (Alzheimer’s disease, vascular dementia, Lewy body dementia, other dementia); neurological – other (multiple sclerosis, seizure disorders or epilepsy, traumatic brain injury (TBI), Parkinson’s disease, stroke/CVA, quadriplegia, paraplegia, hemiplegia, aphasia, motor neuron disease/ALS); intellectual/developmental disability (Down syndrome, autism spectrum disorder, other intellectual disability (organic, non-organic, or cause unknown); cardiac or pulmonary (coronary heart disease, atrial fibrillation, heart failure, peripheral vascular disease (PVD), chronic obstructive pulmonary disease (COPD)); psychiatric (anxiety, bipolar disorder, depression, schizophrenia, post-traumatic stress disorder (PTSD)); infections (pneumonia during last 30 days, urinary tract infection during last 30 days, sepsis during last 30 days, chronic hepatitis (e.g., hepatitis B, C), Clostridium difficile (C. diff), tuberculosis (TB), multidrug resistant organism (MDRO), HIV/AIDS; cancer, diabetes mellitus type 1, type 2, chronic kidney disease (CKD)

**ICD-11 codes of the major diagnoses recorded additionally by assessors in the analyzed interRAI LTC dataset (in the “other diagnoses” section):**

musculoskeletal diagnoses (NC72.2, NC72.Y, NC72.30, FB80.A, NB52.14, NB52.12, NA02.7, NA02.5, NA02.2, NA02.3&XA14T2, NA02.14, NA02.00, NA02.8, NA02.01, NA02.1A&XA2P19, NA02.3, NA02.Y, NA02.Z, ND56.2, FB8Y, ND32, NC53.1&XA06T2, NA22.Z&XA5CQ0, ND13.3, NC53.5, NC53.3, ND13.7, NC53.1&XA9DH2, NC53.6, NC12.0, NC53.1&XA8SZ6, NC53.1&XA7XM2, NC53.4, NC53.1&XA4A64, ND13.2, ND13.0, NC53.2, NC53.1&XA8488, NC53.1, NC53.Y&XA09H2, NC53.0, NC53.2&XA5N95, NC53.2&XA8J87); neurological – dementia (6D80, 6D81, 6D82, 6D8Y); neurological – other (8A40, 8A68.Y, 8A64/4B4Z, 8A64/8E4Y, 8A68.Z, 8A61.1, 8A61.40, 8A60, 8A61.4Y, 8A61.31, 8A61.21, 8A61.20, 8A60.9, 8A61.41, 8A61.30, 8A60.0Y, 8A60.1, 8A60.2, 8A60.7, 8A60.3, 8A6Z, 8A62, 8A61.2Y, 8A61.2, 8A61.2Z, 8A6Y, 8A60.4, 8A60.B, 8A61.0, 8A61.3Y, 8A60.1/8B20, 5C54.Y, LD24.8Y, NA07,8A00.0,8B20, 8B11, 8B26.0&XA5KS6, 8B26.Y, 8B26.1, 8B26.5, 8B26.0&XA9CM4, 8B00, 8B26.4, KB00.0, MB50, MB56, MB53, MA80.0, 8B60); intellectual/developmental disability (LD40.0, 6A02, SD8Y); cardiac or pulmonary (BA8Z, BA8Y, BC81.3, BD1Z, BD1Y, BD1Z&XT5R, BD1Z&XT8W, BD11, BD13, BD10, BD13&XT5R, BD10&XT5R, BD10&XT8W, BD14, BD53.Y, CA22); psychiatric (MB24.3, 6A60, 6A61, 6A7Z, 6A72, 6A7Y, 6A71, 6A70, 6A20, 6B40); infections (CA40, GC08, 1G40, 1G41, 1D9Y/1G40, 1D9Y/1G41, DB97.2, 1E51, 1A1Y&XN0SE, 1A04, 1A04/DB32.2Z, 1B1Z, 1B1Y, 1B13, 1B10, 1B11.Y, 1B12, 1C62), other (2D4Z, 2D42, 2C0Z, 2B93, 2C0Y, 2B50, 2B5K, 2E2Z, QC40, 2B6E, XH0V86, 2C25, 2C90, 2E60.2, 2C12.0, 2C12.0Z, 2A00.5, 2D00, 2D02, 2D02.Z, 5A14, 5A10, 5A11, GB61)

**Supplementary Table 1.** Appropriate maximal single and daily doses of individual BZDs in older patients and the prevalence of the use of higher than recommended doses of BZDs in NH residents

| **BZD** | **ATC**  **code** | **Indication** | **Individual recommended dose** | **Individual max. dose^a^** | **Daily max. dose^a^** | **No. of NH residents exceeding  individual max. dose (% of 126 BZD users)^b^** | | **No. of NH residents exceeding  daily max. dose (% of 126 BZD users)^c^** | |
| --- | --- | --- | --- | --- | --- | --- | --- | --- | --- |
| **Medium acting** | | | | | | | | | |
| alprazolam | N05BA12 | Anxiety | 0.25 mg | 0.25 mg | 0.75 mg | 9 | (7.2%) | 5 | (4.1%) |
| lorazepam | N05BA06 | Anxiety | 1-2 mg | 2 mg | 10 mg | 2 | (1.6%) | 0 | (0.0%) |
| oxazepam | N05BA04 | Anxiety | 10 mg | 15 mg | 60 mg | 0 | (0.0%) | 0 | (0.0%) |
| **Long acting** | | | | | | | | | |
| diazepam | N05BA01 | Anxiety | 2 mg | 2.5 mg | 5 mg | 21 | (16.7%) | 2 | (1.7%) |
|  |  | Muscle spasms | 2 mg | 2.5 mg | 5 mg | 12 | (9.5%) | 1 | (0.8%) |
|  |  | Epilepsy | 4 mg | 2.5 mg | 5 mg | 2 | (1.6%) | 0 | (0.0%) |
| bromazepam | N05BA08 | Anxiety | 1.5 mg | 1.5 mg | 4.5 mg | 2 | (1.6%) | 0 | (0.0%) |
| flurazepam | N05CD01 | Insomnia | 15 mg | 15 mg | 15 mg | 0 | (0.0%) | 0 | (0.0%) |
| nitrazepam | N05CD02 | Insomnia | 2.5 mg | 5 mg | 5 mg | 0 | (0.0%) | 0 | (0.0%) |
|  |  | Epilepsy | 0.05 mg/kg | 0.25 mg/kg | 1 mg/kg | 0 | (0.0%) | 0 | (0.0%) |

*^a^ https://cima.aemps.es/cima/dochtml/ft/56025/FT_56025.html, https://cima.aemps.es/cima/dochtml/ft/68477/FT_68477.html, https://www.drugs.com/dosage/oxazepam.html, https://cima.aemps.es/cima/dochtml/ft/80698/FT_80698.html, https://cima.aemps.es/cima/dochtml/ft/50234/FT_50234.html, https://cima.aemps.es/cima/dochtml/ft/73133/FT_73133.html, http://bijsluiters.fagg-afmps.be/leafletSearchPage.jsf?medicinalProductId=601,* [*http://bijsluiters.fagg-afmps.be/leafletSearchPage.jsf?medicinalProductId=602*](http://bijsluiters.fagg-afmps.be/leafletSearchPage.jsf?medicinalProductId=602)

*Geriatric dose limits were also identified by using these explicit expert panel criteria oN potentially inappropriate geriatric prescribing:*

*[33] 2019 American Geriatrics Society Beers Criteria® Update Expert Panel. American Geriatrics Society 2019 Updated AGS Beers Criteria® for Potentially Inappropriate Medication Use in Older Adults. J Am Geriatr Soc. 2019;67:674–694.*

*[34] O’Mahony D, O’Sullivan D, Byrne S, O’Connor MN, Ryan C, Gallagher P. STOPP/START criteria for potentially inappropriate prescribing in older people: version 2. Age Ageing. 2015;44:213–218.*

*[35] Renom-Guiteras A, Meyer G, Thürmann PA. The EU(7)-PIM list: a list of potentially inappropriate medications for older people consented by experts from seven European countries. Eur J Clin Pharmacol. 2015;71:861–875.^b^ Missing values in individual max. dose: N=125 for N05BA12; ^c^ Missing values in daily max. dose: n=123 for N05BA12, n=125 for N05BA04, n=120 for N05BA01, n=124 for N05BA01 and n=124 for N05BA08;*

**Supplementary Table 2.** Prevalence of the use of BZDs in combination with various medications having sedative properties (classified by ATC subgroup, INN and ATC codes of active substances)

| **Chemical subgroup^a^** | **ATC code (subgroup)** | **International Nonproprietary Name (INN)** | **ATC code (active substance)** | **No. of NH residents using BZDs in combination with medications having sedative properties (% of 126 BZD users)** | |
| --- | --- | --- | --- | --- | --- |
| benzodiazepine related drugs | N05CF* | whole chemical subgroup |  | 20 | (15.9%) |
| other antihistamines for systemic use | R06AX* | whole chemical subgroup |  | 9 | (7.1%) |
| diazepines, oxazepines, thiazepines and oxepines | N05AH | quetiapine | N05AH04 | 8 | (6.3%) |
| butyrophenone derivatives | N05AD | haloperidol | N05AD01 | 5 | (4.0%) |
| other antidepressants | N06AX | mirtazapine | N06AX11 | 3 | (2.4%) |
| benzodiazepine derivatives | N03AE | clonazepam | N03AE01 | 2 | (1.6%) |
| other centrally acting agents | M03BX | baclofen | M03BX01 | 1 | (0.8%) |
| phenothiazines with aliphatic side-chain | N05AA | promazine | N05AA03 | 1 | (0.8%) |
| phenothiazines with piperazine structure | N05AB* | whole chemical subgroup |  | 1 | (0.8%) |
| other antipsychotics | N05AX | aripiprazole | N05AX12 | 1 | (0.8%) |
| non-selective monoamine reuptake inhibitors | N06AA | amitriptyline | N06AA09 | 1 | (0.8%) |
| other antidepressants | N06AX | trazodone | N06AX05 | 1 | (0.8%) |
| piperazine derivatives | R06AE | cetirizine | R06AE07 | 1 | (0.8%) |

*^a^[31] Anatomical Therapeutic Chemical (ATC) Classification [Internet]. [cited 2023 Feb 6]. Available from: https://www.who.int/tools/atc-ddd-toolkit/atc-classification.*

**whole chemical subgroup*

*The list of analyzed sedative medications included ATC codes of sedative drugs (N=248) from different ATC classes summarized in the diploma thesis:*

*Magátová, A. Racionalita užitia benzodiazepínov u starších pacientov (Rationality of benzodiazepines use in older patients). Faculty of Pharmacy in Hradec Králové, Charles University, Czech Republic. Department of Social and Clinical Pharmacy (diploma thesis, defended 05.09.2021. Aleph syst. No:9925470405206986, DSpace syst. No:20.500.11956/170849.*

*When BZD use was analyzed in the context of combinations with fix-dose combined drug including psychotropics, these fix-dose products*

*(including psychotropics) were identified: N02AJ13 tramadol and paracetamol, N02AJ14 tramadol and dexketoprofen, N04BA02 levodopa and decarboxylase inhibitor.*

**Supplementary Table 3.** List of potential drug interactions of BZDs of moderate to strong significance identified in our sample by using MedScape and DrugBank databases

| **Interaction^a^** | **ATC codes** | **Negative effect of the interaction** | **Clinical significance** | **No. of NH residents  (% of 126 BZD users)** | |
| --- | --- | --- | --- | --- | --- |
| alprazolam/diazepam/lorazepam/oxazepam + moxonidine | N05BA12/N05BA01/N05BA06/N05BA04+ C02AC05 | alprazolam/diazepam/lorazepam/oxazepam and moxonidine increase sedation | moderate | 13 | (10.3%) |
| diazepam/nitrazepam + paracetamol | N05BA01/N05CD02 + N02BE01 | diazepam/nitrazepam may increase the hepatotoxic activities of paracetamol | moderate | 3 | (2.4%) |
| alprazolam + metildigoxin | N05BA12 + C01AA08 | alprazolam increases levels of digoxin by an unknown mechanism | moderate | 3 | (2.4%) |
| diazepam + mebeverine | N05BA01 + A03AA04 | diazepam may increase the central nervous system depressant activities of mebeverine | moderate | 2 | (1.6%) |
| diazepam/nitrazepam + levodopa | N05BA01/N05CD02 + N04BA02 | the risk or severity of adverse effects can be increased when diazepam/nitrazepam is combined with levodopa | moderate | 2 | (1.6%) |
| alprazolam + verapamil | N05BA12 + C08DA01 | verapamil will increase the level or effect of alprazolam by affecting hepatic/intestinal enzymes of CYP3A4 metabolism | moderate | 2 | (1.6%) |
| diazepam + propiverine | N05BA01 + G04BD06 | diazepam may increase the central nervous system depressant activities of propiverine | moderate | 1 | (0.8%) |
| diazepam + prednisone | N05BA01 + H02AB07 | prednisone will decrease the level or effect of diazepam by affecting hepatic/intestinal enzyme CYP3A4 metabolism | moderate | 1 | (0.8%) |
| diazepam/nitrazepam + carbamazepine | N05BA01/N05CD02 + N03AF01 | carbamazepine will decrease the level or effect of diazepam/nitrazepam by affecting hepatic/intestinal enzymes of CYP3A4 metabolism | severe | 1 | (0.8%) |
| diazepam/nitrazepam + rasagiline | N05BA01/N05CD02 + N04BD02 | the risk or severity of adverse effects can be increased when diazepam/nitrazepam is combined with rasagiline | moderate | 1 | (0.8%) |
| oxazepam + urapidil | N05BA04 + C02CA06 | the risk or severity of adverse effects can be increased when oxazepam is combined with urapidil | moderate | 1 | (0.8%) |
| broamzepam + levocetirizine | N05BA08 + R06AE09 | the risk or severity of adverse effects can be increased when bromazepam is combined with levocetirizine | moderate | 1 | (0.8%) |
| bromazepam/diazepam + cinnarizine | N05BA08/N05BA01 + N07CA02 | the metabolism of bromazepam can be decreased when combined with cinnarizine; cinnarizine and diazepam both increase sedation | moderate | 1 | (0.8%) |
| alprazolam + metronidazole | N05BA12 + P01AB01 | metronidazole will increase the level or effect of alprazolam by affecting hepatic/intestinal enzymes of CYP3A4 metabolism | moderate | 1 | (0.8%) |
| alprazolam + pholcodine | N05BA12 + R05DA08 | alprazolam and pholcodine both increase sedation | moderate | 1 | (0.8%) |
| alprazolam/diazepam + darifenacin | N05BA12/N05BA01 + G04BD10 | darifenacin will increase the level or effect of alprazolam/diazepam by affecting hepatic/intestinal enzymes of CYP3A4 metabolism | moderate | 1 | (0.8%) |
| nitrazepam + oxcarbazepine | N05CD02 + N03AF02 | the risk or severity of adverse effects can be increased when oxcarbazepine is combined with nitrazepam | moderate | 1 | (0.8%) |
| alprazolam/nitrazepam + pregabalin | N05BA12/N05CD02 + N02BF02 | pregabalin and alprazolam/nitrazepam increase the effects of the other drug by pharmacodynamic synergism; coadministration of CNS depressants can result in serious, life-threatening, and fatal respiratory depression; the therapeutic efficacy of nitrazepam can be increased when used in combination with pregabalin | moderate | 0 | (0.0%) |

*^a^[36] Drug Interactions Checker - Medscape Drug Reference Database [Internet]. [cited 2023 Feb 20]. Available from: https://reference.medscape.com/drug-interactionchecker*

*[37] Drug Interaction Checker | DrugBank Online [Internet]. [cited 2023 Feb 20]. Available from: https://go.drugbank.com/drug-interaction-checker#results.*

**Supplementary Table 4.** Prevalence of the use of BZDs in combination with medications having anticholinergic properties

| **International Nonproprietary Name (INN)** | **ATC code** | **Anticholinergic activity** | **No. of NH residents**  **(% of 126 BZD users)** | |
| --- | --- | --- | --- | --- |
| diazepam | N05BA01 | 1 | 67 | (53.2%) |
| tramadol and paracetamol | N02AJ13 | 1 | 51 | (40.5%) |
| furosemide | C03CA01 | 1 | 45 | (35.7%) |
| alprazolam | N05BA12 | 1 | 36 | (28.6%) |
| oxazepam | N05BA04 | 1 | 20 | (15.9%) |
| metformin | A10BA02 | 1 | 16 | (12.7%) |
| warfarin | B01AA03 | 1 | 9 | (7.1%) |
| quetiapine | N05AH04 | 1 | 8 | (6.3%) |
| isosorbide mononitrate | C01DA14 | 1 | 7 | (5.6%) |
| sertraline | N06AB06 | 1 | 7 | (5.6%) |
| bromazepam | N05BA08 | 1 | 6 | (4.8%) |
| ipratropium bromide | R03BB01 | 2 | 6 | (4.8%) |
| lorazepam | N05BA06 | 1 | 6 | (4.8%) |
| sulpiride | N05AL01 | 1 | 6 | (4.8%) |
| haloperidol | N05AD01 | 1 | 5 | (4.0%) |
| isosorbide dinitrate | C01DA08 | 1 | 5 | (4.0%) |
| loratadine | R06AX13 | 1 | 4 | (3.2%) |
| metoclopramide | A03FA01 | 1 | 4 | (3.2%) |
| mirtazapine | N06AX11 | 1 | 3 | (2.4%) |
| theophylline | R03DA04 | 1 | 3 | (2.4%) |
| tramadol | N02AX02 | 1 | 3 | (2.4%) |
| clonazepam | N03AE01 | 1 | 2 | (1.6%) |
| escitalopram | N06AB10 | 1 | 2 | (1.6%) |
| fexofenadine | R06AX26 | 1 | 2 | (1.6%) |
| mebeverine | A03AA04 | 1 | 2 | (1.6%) |
| salmeterol and fluticasone | R03AK06 | 1 | 2 | (1.6%) |
| solifenacin | G04BD08 | 2 | 2 | (1.6%) |
| amantadine | N04BB01 | 2 | 1 | (0.8%) |
| amitriptyline | N06AA09 | 3 | 1 | (0.8%) |
| aripiprazole | N05AX12 | 1 | 1 | (0.8%) |
| baclofen | M03BX01 | 1 | 1 | (0.8%) |
| carbamazepine | N03AF01 | 1 | 1 | (0.8%) |
| cetirizine | R06AE07 | 1 | 1 | (0.8%) |
| cinnarizine | N07CA02 | 1 | 1 | (0.8%) |
| darifenacin | G04BD10 | 2 | 1 | (0.8%) |
| desloratadine | R06AX27 | 1 | 1 | (0.8%) |
| fluphenazine | N05AB02 | 2 | 1 | (0.8%) |
| flurazepam | N05CD01 | 1 | 1 | (0.8%) |
| Fluvoxamine | N06AB08 | 1 | 1 | (0.8%) |
| levocetirizine | R06AE09 | 1 | 1 | (0.8%) |
| oxcarbazepine | N03AF02 | 2 | 1 | (0.8%) |
| paracetamol, combinations excl. psycholeptics | N02BE51 | 1.5 | 1 | (0.8%) |
| paroxetine | N06AB05 | 2 | 1 | (0.8%) |
| prednisone | H02AB07 | 1 | 1 | (0.8%) |
| promazine | N05AA03 | 3 | 1 | (0.8%) |
| propiverine | G04BD06 | 3 | 1 | (0.8%) |
| rasagiline | N04BD02 | 1 | 1 | (0.8%) |
| trazodone | N06AX05 | 1 | 1 | (0.8%) |

*[31] Anatomical Therapeutic Chemical (ATC) Classification [Internet]. [cited 2023 Feb 6]. Available from: <https://www.who.int/tools/atc-ddd-toolkit/atc-classification>.*

*The whole list of analyzed ACH medications was based on the comprehensive list of active substances (n=838) identified from previously validated ACH drug burden scales (published by 2022). These were specifically: Clinician-rated Anticholinergic Score (Cr-ACHS) [Han et al. Arch Intern Med. 2001; Han et al. J Am Geriatr Soc. 2008], Anticholinergic Drug Scale (ADS) [Carnahan et al. Psychopharmacol Bull. 2002; Carnahan et al. J Clin Pharmacol. 2006], Anticholinergic Burden Classification (ABC) [Ancelini et al. BMJ, 2006], Cancelli’s Anticholinergic Burden Scale (CABS) [Cancelli et al. Clin Pharmacol Ther. 2008], Anticholinergic Risk Scale (ARS) [Rudolph. Arch Intern Med, 2008], Anticholinergic Cognitive Burden Scale (ACB) [Boustani et al. Aging health. 2008; Campbell et al. J Am Geriatr Soc. 2013], Chew’s List/Chew’s scale [Chew et al. J Am Geriatr Soc. 2008], Anticholiergic Activity Scale (AAS) [Ehrt et al. J Neurol Neurosurg Psychiatry. 2010], Anticholinergic Loading Scale (ACL/ALS) [Sittironnarit et al. Dementia and Geriatric Cognitive Disorders. 2011], Delirogenic Risk Scale (DRS) [Hefner et al. J J Pharma Pharmacovigilance. 2015], Anticholinergic Effect on Cofnition (AEC) [Bishara et al. Int J Geriatr Psychiatry. 2017] and Korean Anticholinergic Burden Scale (KABS) [Jun et al. Geriatr Gerontol Int. 2019].*

*Among 226 residents in our study, 45 (19.9%), 48 (21.2%), 45 (19.9%), 88 (38.9%) were using drug regimens with anticholinergic activity 0, 1, 2, >2, respectively. Among 126 BZD users, 2 (1.6%), 22 (17.5%), 27 (21.4%), 75 (59.5%) were using drug regimens with anticholinergic activity 0, 1, 2, >2, respectively.*
